# Research on Soft Rock Damage Softening Model and Roadway Deformation and Failure Characteristics

**DOI:** 10.3390/ma15175886

**Published:** 2022-08-26

**Authors:** Chunlin Zeng, Yuejin Zhou, Yuhang Xiao, Xin Zhou, Chaobin Zhu, Yunong Xu

**Affiliations:** State Key Laboratory for Geomechanics & Deep Underground Engineering, China University of Mining & Technology, Xuzhou 221116, China

**Keywords:** soft rock roadway, damage softening model, stability analysis

## Abstract

To determine a reasonable control strategy for deep buried soft rock roadways, a study on deformation and failure characteristics was carried out. The Weibull distribution damage variable was introduced to construct a damage-softening model considering the lateral deformation of the rock mass, and the functional relationship between the model parameters *F*_0_ and *m* and the confining pressure were discussed. The nonlinear fitting method was used to correct the model parameters. Using the model, the failure characteristics of deep buried soft rock roadways were analyzed. A comprehensive and step-by-step joint support control strategy was proposed based on the numerical simulation results. The research results showed that the damage-softening model curve established could genuinely reflect the whole process of mudstone failure. The apparent stress concentration phenomenon occurred in the surrounding rock. The surrounding rock deformation showed that roadway floors had larger plastic failure areas than sides and vaults. The plastic failure depth could reach 2.45 m. After a comprehensive and step-by-step joint support control strategy was adopted, the deformation rate of the roadway at the section was less than 0.1 mm/d. The optimized support scheme can effectively improve the stability of the roadway.

## 1. Introduction

Coal has long been China’s primary energy source. With the gradual mining of deep coal resources, the number of deep buried soft rock roadway examples has increased [1,2,3]. Because the deep rock mass has been stored in a complex, high-stress state for a long time, its mechanical properties have changed drastically, and the deformation failure mode is quite different from that of the shallow part [4,5]. At the same time, soft rock has prominent mechanical deterioration characteristics under the action of external force disturbance and high in situ stress [6,7,8]. Therefore, in the deep environment of “the three highs and the one disturbance”, the resulting roadway can experience rock instability and support structure failure. Such engineering problems seriously restrict safe and efficient production of mines [9,10,11]. Therefore, a study into the mechanical properties of deep soft rock is critical for guiding the stability management of the surrounding rock.

In recent years, the failure and deformation mechanism, instability, and support methods of soft rock roadways have received attention and extensive research. Wang et al. [12] carried out numerical tests comprehensively considering arch strength, in situ stress, and surrounding rock mechanical parameters; determined the damage and control mechanism of the deep soft rock roadway; and proposed the concept of “integrity, high strength, and decompression”. According to numerical simulation calculations and field tests, Li et al. [7] analyzed the mode, influencing factors, laws, and mechanisms of soft rock roadway deformation and failure and put forward the combined support method of “double-layer long anchor mesh shotcrete and concrete-filled steel pipe”. For studying control methods of soft rock roadways, Zhao et al. [13] proposed the composite support method of “shotcrete + grouting bolt + anchor bolt + grouting anchor cable + anchor cable” according to the soft rock characteristics of the surrounding rock. Chen et al. [14] studied the control method of the surrounding rock using theoretical analysis and numerical simulation. Yang et al. [15] used UDEC to simulate the failure process of the roadway under the condition of primary support and analyzed the stress and deformation of the surrounding rock. Many scholars have studied the failure mechanism of deep soft rock roadways through field experiments, physical model tests, numerical simulations, and theoretical analysis [16,17,18,19]. However, most of these research results only considered the natural strength of soft rocks, ignoring the fact that soft rocks suffer from strain-softening problems such as reduced failure strength and volume expansion [20,21,22,23]. The latter is often the main reason for the continuous expansion of the soft rock roadway deformation and the failure of the supporting structure.

For the mechanical deterioration characteristics of soft rock, many researchers have studied its mechanical evolution characteristics. Yang et al. [24] conducted experimental studies on the mechanical properties of single-stage and multi-stage compression for soft rocks with different damage degrees. Yu et al. [25] established a binary medium model based on triaxial compression experiments of mudstone under different confining pressures and dip angles. Iyare et al. [26] conducted experimental research on mudstone under high-pressure conditions and established a failure behavior model based on the failure of mudstone. Jing et al. [27] studied the creep failure mechanism of mudstone based on micro–mesoscopic experiments and numerical simulation of mudstone. Zhang et al. [28] researched the rheological properties and a rheological model of soft rock. However, the above research studies mainly involved analyses of uniaxial and triaxial test results of soft rock, as well as research on the evolution law of rock damage under certain conditions. Only a few research studies involved the nonlinear characteristics and damage mechanics model of the soft rock itself, as well as nonlinear finite element solutions.

Given the above problems, this paper takes a roadway in the Pingdingshan mining area as an example and studies the strain-softening characteristics of mudstone and the evolution law of mechanical parameters through indoor triaxial compression tests. The Weibull distribution damage variable is introduced. The distribution parameters are determined based on the test results to construct a damage-softening model considering lateral deformation of the rock mass. FLAC3D (6.0, Minneapolis, MN, USA) is used to solve the damage-softening model and verify it. The numerical calculation model of the surrounding rock is established, and its stress and deformation are analyzed. Moreover, on this basis, according to the damage characteristics, a comprehensive and step-by-step joint support control strategy is proposed. The research results are significant for ensuring the stability of deeply buried, weakly cemented soft rock roadways and for realizing safe and efficient mine production.

## 2. Establishment of a Soft Rock Damage and Softening Model

### 2.1. Definition of Damage Variables

Considering that soft rock is composed of countless micro-elements, the following assumptions are made regarding soft-rock micro-elements [29]: (1) all sides of the micro-element are equal; (2) the geometric characteristics of the micro-element are isotropic; (3) the microelement is isotropic; and (4) the element is small enough to be considered a continuous medium. Setting the element strength F depends on its own failure criterion, and the Drucker–Prager failure criterion [30] is used as the element strength:

(1) $$F=\alpha_{0}I_{1}+\sqrt{J_{2}}$$

where *α*_0_ is a parameter related to internal friction angle *φ*; and *I*_1_ and *J*_2_ are the first and second stress tensor invariant, respectively. These parameters are calculated as follows:

(2) $$\alpha_{0}=\frac{\sin\varphi }{\sqrt{9+3\;\sin^{2}\varphi }}$$

(3) $$I_{1}=\sigma_{1}+\sigma_{2}+\sigma_{3}$$

(4) $$\sqrt{J_{2}}=\sqrt{{\left(\sigma_{1}-\sigma_{2}\right)}^{2}+{\left(\sigma_{2}-\sigma_{3}\right)}^{2}+{\left(\sigma_{3}-\sigma_{1}\right)}^{2}}/\sqrt{6}$$

where *σ*_i_ (i = 1, 2, 3) is the rock’s principal stresses in the i-th direction under triaxial compression.

Due to the existence of the condition *σ*_2_ = *σ*_3_ in the conventional triaxial compression test, we can obtain:

(5) $$I_{1}=\frac{\left(\sigma_{1}+2\sigma_{3}\right)E\varepsilon_{1}}{\sigma_{1}-2\mu \sigma_{3}}$$

(6) $$\sqrt{J_{2}}=\frac{\left(\sigma_{1}-\sigma_{3}\right)E\varepsilon_{1}}{\sqrt{3}\left(\sigma_{1}-2\mu \sigma_{3}\right)}$$

where *ε*_i_ (i = 1, 2, 3) is the rock’s principal strain in the i-th direction under triaxial compression; and *µ* is the Poisson ratio of the rock sample.

Substituting Equations (2), (5), and (6) into Equation (1), the microelement intensity is obtained:

(7) $$F=\frac{\sin\varphi }{\sqrt{9+3\;\sin^{2}\varphi }}\cdot \frac{\left(\sigma_{1}+2\sigma_{3}\right)E\varepsilon_{1}}{\sigma_{1}-2\mu \sigma_{3}}+\frac{\left(\sigma_{1}-\sigma_{3}\right)E\varepsilon_{1}}{\sqrt{3}\left(\sigma_{1}-2\mu \sigma_{3}\right)}$$

Due to the severe heterogeneity of the rock, the micromechanical behavior is difficult to quantify, and the damage caused by external loads can be deduced by mathematical statistics methods. The rock micro-element strength is set as a randomly distributed variable, which fits the Weibull distribution function [20,21], so the probability density function is obtained as follows:

(8) $$P(F)=\frac{m}{F_{0}}{\left(\frac{F}{F_{0}}\right)}^{m-1}\exp\left[-{\left(\frac{F}{F_{0}}\right)}^{m}\right]$$

where *P* is the probability density; *m* and *F*_0_ are the Weber distribution parameters; and *F* is the Weibull distribution variable of the mudstone microelement yield.

Since the rock is considered to be a micro-scale continuum, the damage variable *D* is derived by dividing the number of the damaged micro-elements *N_D_* by the number of all the micro-elements *N*:

(9) $$D=\frac{N_{D}}{N}$$

In the random interval [*F*, *F* + *dF*], the damage element *N_D_* is equivalent to *NP* (*F*) *dF*, which can be obtained under a certain plastic strain:

(10) $$N_{D}=\int_{0}^{F}NP\left(F\right)dF=N\left\{1-\exp\left[-{\left(\frac{F}{F_{0}}\right)}^{m}\right]\right\}$$

The damage variable *D* input into Equation (10) yields:

(11) $$D=1-\exp\left[-{\left(\frac{F}{F_{0}}\right)}^{m}\right]$$

### 2.2. Establishment of Damage Softening Construct

There are two kinds of rock microelements: one part that is undamaged, and one part that is damaged. The damaged part has no bearing capacity. Therefore, according to Hooke’s law and the principle of effective stress [6], the normal stress of the microelement is:

(12) $$\sigma_{i}=\sigma_{i}^{*}(1-D)$$

According to the above assumptions, the deformation of undamaged rock microelements conforms to Hooke’s law. Based on the principle of coordinated deformation, the equation for calculating normal stress is:

(13) $$\sigma_{i}=E\varepsilon_{i}(1-D)+\mu (\sigma_{j}+\sigma_{k})$$

Under conventional triaxial stress conditions (*σ*_2_ = *σ*_3_), the stress-strain relationship is determined as follows:

(14) $$\sigma_{1}=E\varepsilon_{1}(1-D)+2\mu \sigma_{3}$$

(15) $$\sigma_{3}=E\varepsilon_{3}(1-D)+\mu (\sigma_{1}+\sigma_{3})$$

Thus, we can obtain the following:

(16) $$\sigma_{1}-\sigma_{3}=\frac{E(1-D)(\varepsilon_{1}-\varepsilon_{3})}{1+\mu }$$

According to Equations (7), (11), and (16), the damage-softening property constitutive of rock can be obtained as follows:

(17) $$\sigma_{1}=\frac{E}{1+\mu }(\varepsilon_{1}-\varepsilon_{3})\exp\left[-{\left(\frac{F}{F_{0}}\right)}^{m}\right]+\sigma_{3}$$

## 3. Model Validation

### 3.1. Test Plan and Result Analysis

**Figure 1:**
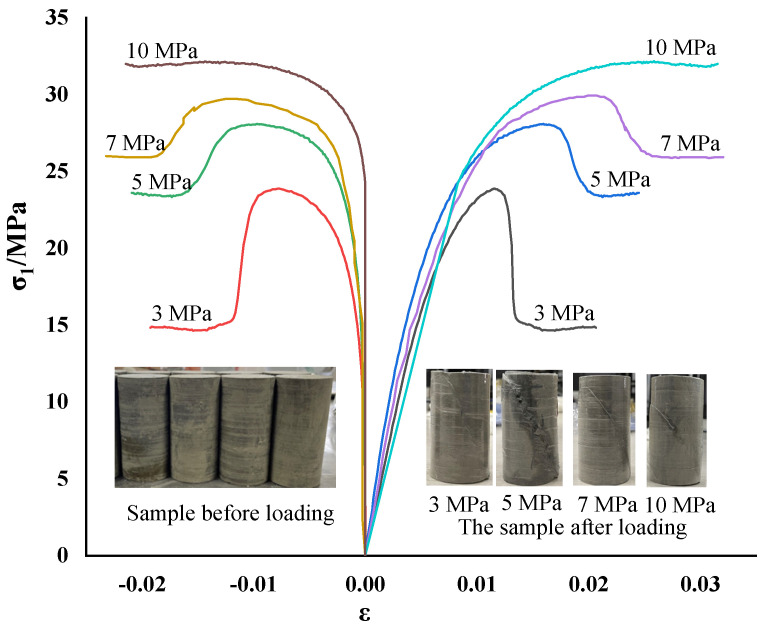
Mudstone stress–strain curves with different confining pressures.

The mudstone samples had uniform texture and good overall integrity and were collected from the roadway of the Pingdingshan mining area in Henan. According to ISRM (International Society for Rock Mechanics) standards, the rock blocks were sectioned and ground to obtain the standard size of Φ50 mm × 100 mm. Rock triaxial testing was carried out on an MTS815 machine (Mechanical Testing and Simulation, Saint Paul, MN, USA). In the mudstone triaxial test, the confining pressure was selected as four grades of 3, 5, 7, and 10 MPa, the displacement control method was adopted, and loading was carried out at a rate of 0.2 mm/min until the pressure was relieved after the sample failed. It was found through a large number of tests that the stress–strain curves of mudstone were similar, so only a representative total stress–strain curve of the mudstone triaxial compression test is shown in Figure 1.

**Figure 2:**
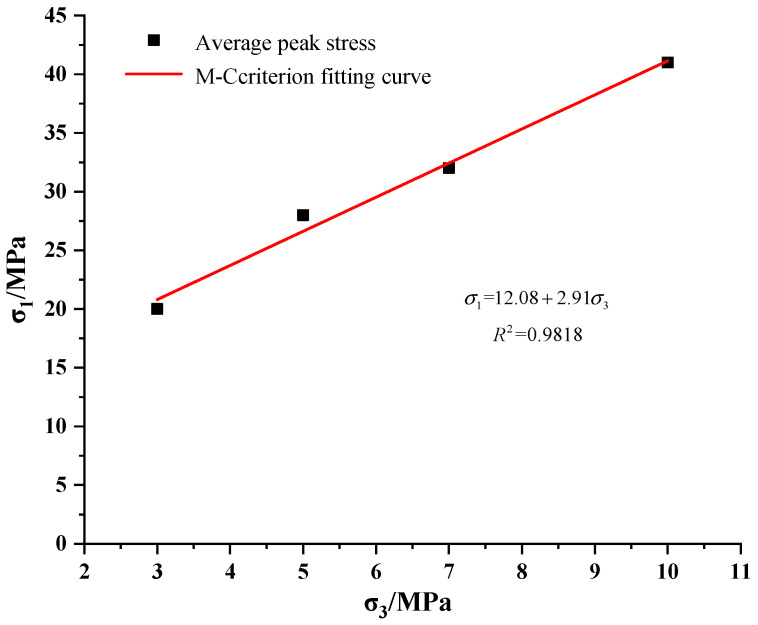
Peak intensity fitting curve.

Figure 1 shows that the damage to mudstone was accompanied by the compaction, cracking, expansion, and other damage processes of the internal fractures of the rock, and the mudstone showed obvious softening characteristics. The main failure mode of mudstone was shear failure. When the test confining pressure was small, the cracks of the rock sample fully expanded, and the phenomenon of obvious volume expansion and expansion occurred. With the rise of the test confining pressure, the failure shear zone of the rock sample became more obvious, and the confining pressure effectively limited the lateral deformation of the rock sample. Under the different test confining pressure conditions, the stress–strain curve of mudstone had roughly the same development law before reaching the peak value. After reaching the peak stress, the strength gradually decreased, showing obvious strain-softening characteristics. According to the obtained mean value of mudstone peak stress, the fitted mudstone peak strength principal stress curve is shown in Figure 2.

### 3.2. Parameter Correction of Mudstone Damage and Softening Model

**Figure 3:**
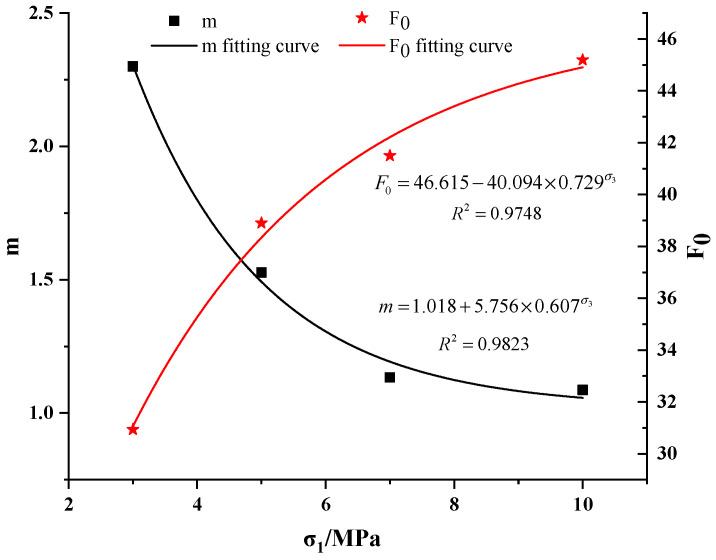
Scatter distribution of *m*–*σ*_3_ and *F*_0_–*σ*_3_

Using the experimental data, the damage variable was obtained from Equation (11), and the parameters *m* and *F*_0_ of Weibull distribution were calculated according to the calculation method of Ren et al. [8]. The model parameters of the obtained mudstone specimen are shown in Table 1. By analyzing the parameters of the Weibull distribution damage softening model of mudstone under triaxial compression, it was established that Weibull distribution parameters (*m* and *F*_0_) had a relationship with the confining pressure *σ*_3_, and then the parameters of the Weibull distribution damage-softening model were modified. As shown in Figure 3, based on the fitting relationship between *m*–*σ*_3_ and *F*_0_–*σ*_3_, the corrected *m* and *F*_0_ parameters were obtained, as shown in Equation (18).

(18) $$\left\{\begin{array}{l} F_{0}=46.615-40.094\times 0.729^{\sigma_{3}}\\ m=1.018+5.756\times 0.607^{\sigma_{3}} \end{array}\right.$$

### 3.3. Validation of the Mudstone Damage and Softening Model

**Figure 4:**
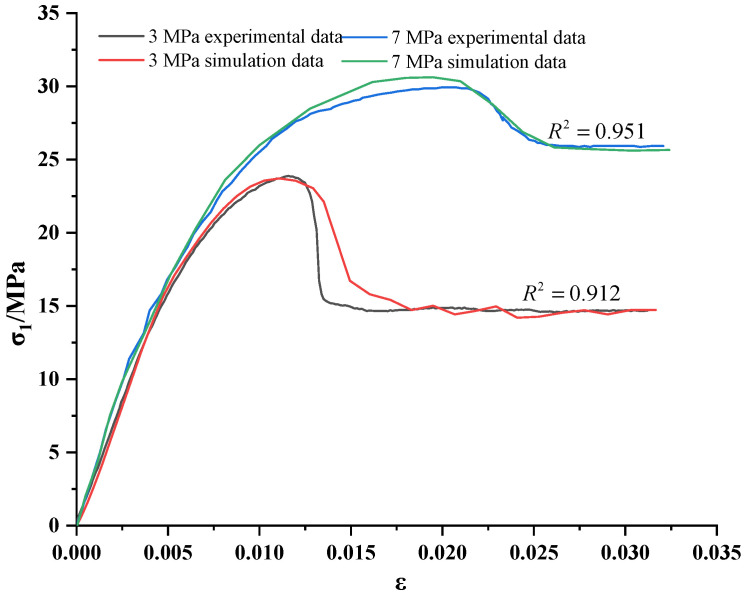
Comparison of numerical and experimental curves for confining pressures of 3 MPa and 7 MPa.

Based on the stress–strain relationship and experimental mechanical parameters of the mudstone damage and softening model, the constitutive model was conducted using the correlation function in the FISH language, and the Mohr–Coulomb criterion was embedded. According to the analysis steps of FLAC3D, the triaxial compression of mudstone under different confining pressures was numerically analyzed. A comparison between the calculated and experimental values of initial confining pressures of 7 MPa and 3 MPa is shown in Figure 4. It can be seen that in the elastic stage and the buckling stage, the numerical calculation curve has the same trend as the test value, and the numerical calculation strength under different confining pressure conditions is relatively close to the test peak strength. The model proposed in this paper could simulate the axial stress–strain behavior well under the condition of mudstone damage and softening and could well reflect the strain-softening process of mudstone.

## 4. Analysis of Deformation and Failure Characteristics of Deeply Buried Soft Rock Roadway

### 4.1. Project Overview

**Figure 5:**
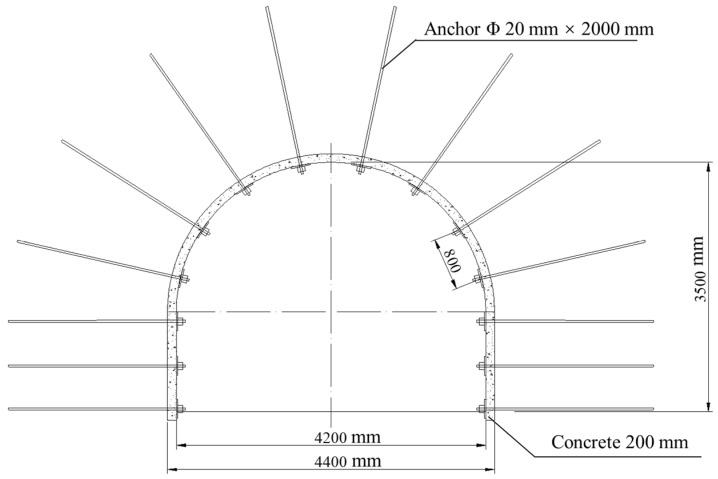
Roadway support diagram.

The Shangshan roadway in the Pingdingshan mining region of Henan Province is 650 m in length. The surrounding rocks are dominated by mudstone. The cross-sectional shape is a straight wall and a semi-circular arch. The roadway cross-section is 4400 mm × 3500 mm. Through an on-site in situ stress test, its vertical self-weight stress and horizontal tectonic stress were found to be about 16.05 MPa and 24.10 MPa, which represent high in situ stress conditions. The initial support of the roadway is composed of bolt and metal mesh supports, the bolts are left-threaded steel bolts with diameters of Φ 20 mm × 2000 mm, and the row spacing is 800 × 800 mm. The top and bottom anchor nets are not less than 100 mm, and the interval is 200–300 mm with wire mesh; 200 mm thick concrete is laid all around the roadway. The original support diagram of the Shangshan roadway is shown in Figure 5. Due to the obvious softening characteristics of mudstone, the roadway has the characteristics of large deformation and instability, which is manifested in the convergence of the roof and floor of the roadway and the two gangs, and there are damages such as bolt breaking, pallet falling, metal mesh shearing, and net pockets.

### 4.2. Numerical Model Establishment and Parameter Setting

**Figure 6:**
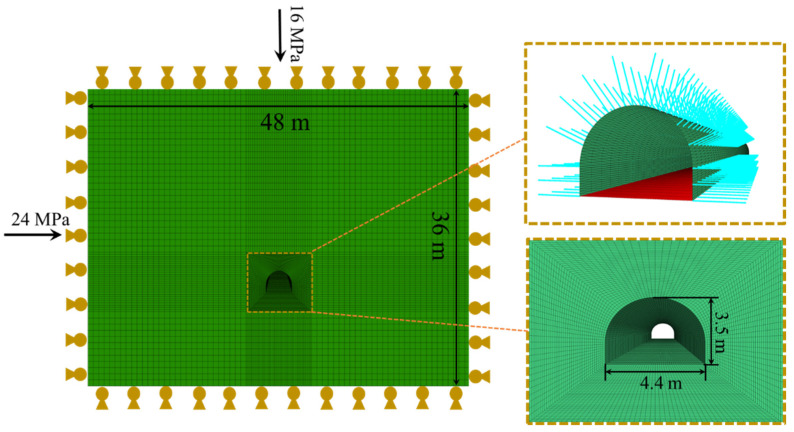
Numerical support model.

According to the geological conditions of the surrounding rock of the roadway and the form of the supporting structure, using the established mudstone damage and softening model, the established FLAC3D numerical model is shown in Figure 6. The horizontal in situ stress and vertical stress were set to 24.0 MPa and 16.0 MPa, respectively. After the initial stress of the model was completed, the simulation of roadway excavation began. During the excavation process, it simulated the bolt with the cable element and simulated the mesh shotcrete and concrete floor with the shell element while setting the mechanical parameters. The mechanical and physical parameters of the roadway support structure are shown in Table 2.

### 4.3. Analysis of Numerical Calculation Results

**Figure 7:**
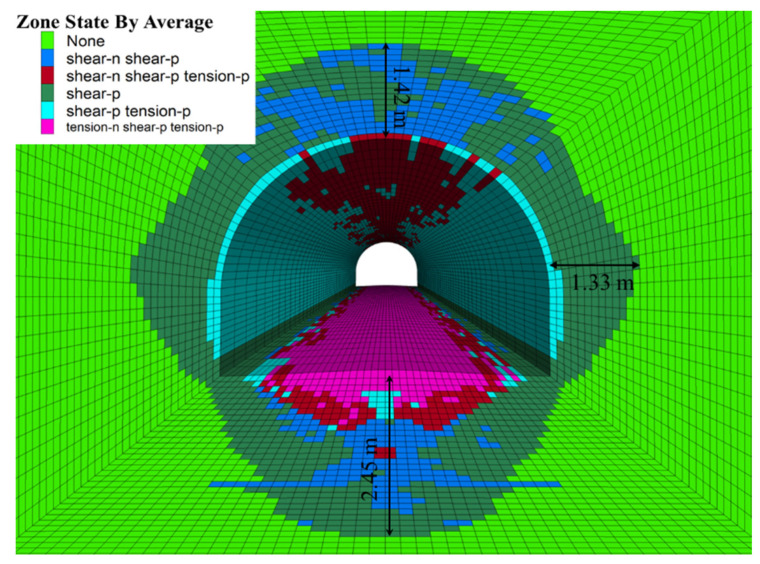
Distribution map of plastic failure area.

As shown in Figure 7, the plastic failure depth of the surrounding rock was 2.45 m. This is because the surrounding rock produced a local shear failure zone under the effect of self-weight stress, and then the local shear failure zones were connected and penetrated into each other, thus forming a large area of the plastic damage zone. Furthermore, due to the large horizontal stress on the roadway, the roadway floors had a larger plastic failure area than the sides and vault.

**Figure 8:**
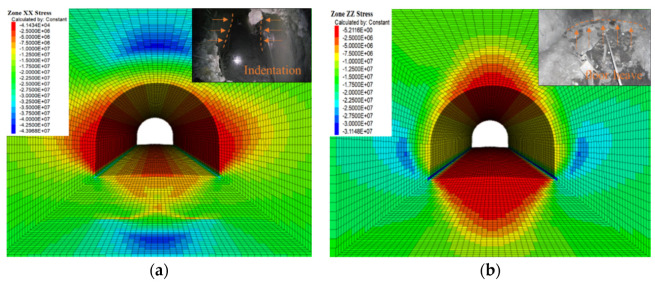
Stress cloud map of surrounding rock: (**a**) horizontal stress contour; (**b**) vertical stress contour.

The surrounding rock stress is shown in Figure 8. When the roadway was excavated under high ground stress, the roadway’s inner side was damaged to varying degrees, indicating that the original support scheme could not effectively restrain the surrounding rock of the deep buried weakly cemented soft rock roadway. The plastic expansion caused the damage degree of surrounding rock to become more serious as the roadway advanced and finally loosened and became unstable. Comparing the numerical calculation results with the actual roadway engineering surrounding rock deformation and damage, it can be seen that the established damage softening model is more reasonable for the numerical simulation analysis of surrounding rock plastic damage zone and support structure stress. It can more realistically reflect the failure characteristics of rock mass and predict the position of the failure area in the surrounding rock of the chamber, and the calculation results can provide scientific guidance for construction.

### 4.4. Optimization of Roadway Support Scheme

Based on the numerical analysis results under the damage softening model and the damage form of the support structure, a comprehensive step-by-step joint support was taken to optimize the roadway. “Grouting bolt + grouting anchor cable + high-strength prestressed anchor cable + steel mesh surface + compound spray layer” was the secondary support, and, finally, the floor grouting + floor anchor cable was applied. The stress state of the surrounding rock was restored in time through one-time support, and the preliminary control of roadway stability and construction safety was realized. The damaged area was repaired by grouting reinforcement support, and the surrounding rock was further strengthened. The stress was realized through anchor cable support transfer and expanded the bearing circle, further improving and restoring the stress conditions of the surrounding rock and realizing the fundamental control of roadway stability.

**Figure 9:**
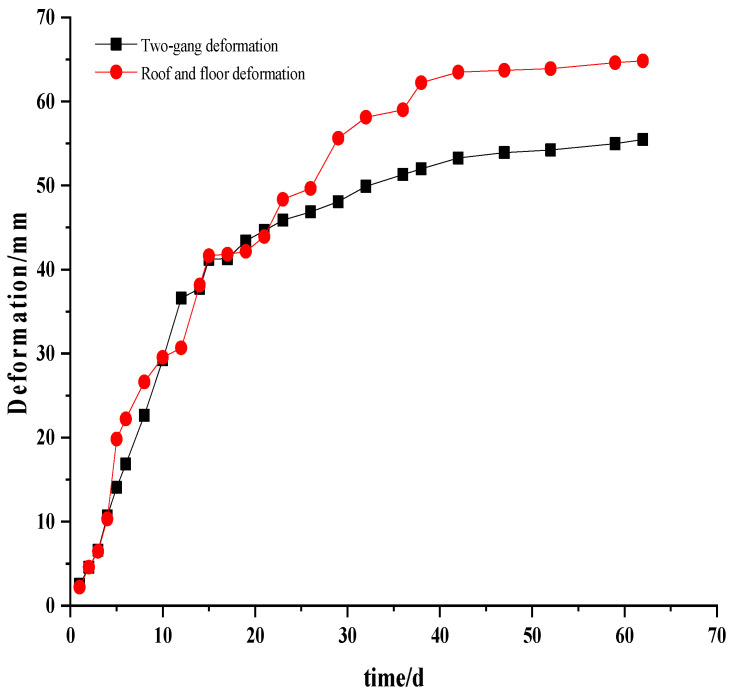
Surrounding rock deformation–time relationship curve.

After the structure had been supported, as shown in Figure 9, due to the influence of the excavation disturbance and the release of its stored deformation energy, the deformation of the surface rock mass increased sharply; after 30 days of excavation, the surface displacement and deformation rate began to decrease, and after 50 to 60 days, the deformation rate was stable. Below 0.1–0.3 mm/d, the overall deformation was within the control range. The onsite monitoring results showed that the optimized roadway support structure had a high safety reserve and a reasonable design scheme.

## 5. Conclusions

(1) The rock is considered to be composed of numerous microelements, which obey isotropic and continuous conditions. Assuming that the rock microelement strength obeys Weibull distribution, based on the principle of statistical damage mechanics and coordinated deformation, a damage-softening model considering lateral deformation of the rock mass is constructed. The structure was optimized, and FLAC3D was used to determine that the model curve was consistent with the experimental curve.

(2) The numerical simulation results based on the mudstone damage-softening model show that local shear failure zones are first generated in surrounding rock under high self-weight stress. Then the local shear failure zones are connected and penetrate each other and gradually form the plastic damage area with a large area; the plastic damage depth of the roadway is 2.45 m. The deformation and softening characteristics of surrounding rock are very significant, showing that the roadway floors have a larger plastic failure area than do the sides and vault.

(3) After adopting the comprehensive and step-by-step joint support control strategy, the deformation rate of the surrounding rock of the section is below 0.1 mm/d, and the two curves of horizontal convergence and roof and floor deformation tend to be stable. The optimized support scheme effectively suppresses the development of the plastic area, reduces the scope of the shear failure area, and effectively improves the stability of the surrounding rock structure.

## Figures and Tables

**Table 1:** Mudstone damage softening constitutive parameters.

*σ*_3_/MPa	c/MPa	*φ*/°	*E*/GPa	*µ*	*m*	*F*_0_
3	2.60	35.06	3.383	0.28	2.2997	30.936
5					1.5271	38.898
7					1.1341	41.498
10					1.0869	45.192

**Table 2:** Support parameters.

Type	*ρ*/(kg·m^–3^)	*E*/GPa	ν	c/MPa	*φ*/(°)
Anchor	7800	215	0.30	0.8	31
Mesh shotcrete	2450	26	0.25		
Concrete floor	2400	30	0.20		

## Data Availability

The data presented in this study are available upon request from the corresponding author.
